# Structural Analysis of Inhibitor Binding to Enterovirus-D68 3C Protease

**DOI:** 10.3390/v17010075

**Published:** 2025-01-08

**Authors:** Vincent N. Azzolino, Ala M. Shaqra, Akbar Ali, Nese Kurt Yilmaz, Celia A. Schiffer

**Affiliations:** Department of Biochemistry and Molecular Biotechnology, University of Massachusetts Chan Medical School, Worcester, MA 01605, USA; vincent.azzolino@umassmed.edu (V.N.A.); ala.shaqra@umassmed.edu (A.M.S.); akbar.ali@umassmed.edu (A.A.); nese.kurtyilmaz@umassmed.edu (N.K.Y.)

**Keywords:** enterovirus, EV68, protease, drug resistance, protein structure, inhibition assays

## Abstract

Enterovirus-D68 (EV68) continues to present as a global health issue causing respiratory illness and outbreaks associated with long-lasting neurological disease, with no antivirals or specific treatment options. The development of antiviral therapeutics, such as small-molecule inhibitors that target conserved proteins like the enteroviral 3C protease, remains to be achieved. While various 3C inhibitors have been investigated, their design does not consider the potential emergence of drug resistance mutations. For other antivirals where resistance has been a challenge, we have demonstrated that the likelihood of resistance can be minimized by designing inhibitors that leverage the evolutionary constraints of the target. Here, we characterize a series of 3C inhibitors against EV68-3C protease through enzyme inhibition, protein crystallography, and structural analysis. We have determined and analyzed three high-resolution inhibitor-bound crystal structures of EV68-3C protease, which revealed possible sites of resistance mutations, a key structural water molecule conserved during ligand binding, and the conformational flexibility of the catalytic histidine H40. This structural analysis combined with enzymatic assays provides insights for the rational design of inhibitors that are robust against resistance toward developing antiviral treatments for EV68 infections.

## 1. Introduction

The Picornaviridae family of single-stranded, positive-sense, non-enveloped RNA viruses includes several pathogens of concern such as enterovirus-D68 (EV68) [1]. EV68 primarily infects infants, children, and immunocompromised individuals [1,2], and, while most infections are self-limiting, EV68 infections can cause severe respiratory symptoms as well as devastating and long-lasting neurological deficits [2,3,4,5]. EV68 has been associated with acute flaccid myelitis (AFM) [6,7], a polio-like paralysis with debilitating muscle weakness [8], where more than 90% of patients never reach full neurologic recovery [5,8,9,10]. Outbreaks across the United States in 2014, 2016, and 2018 caused reported cases in the thousands with severe respiratory illness, the death of dozens of children, and the increased incidence of AFM associated with EV68 [2,6,11,12,13,14]. The trend of outbreaks for this neurotropic strain of enterovirus is cause for global concern and highlights the need to develop effective therapeutics.

**Figure 1:**
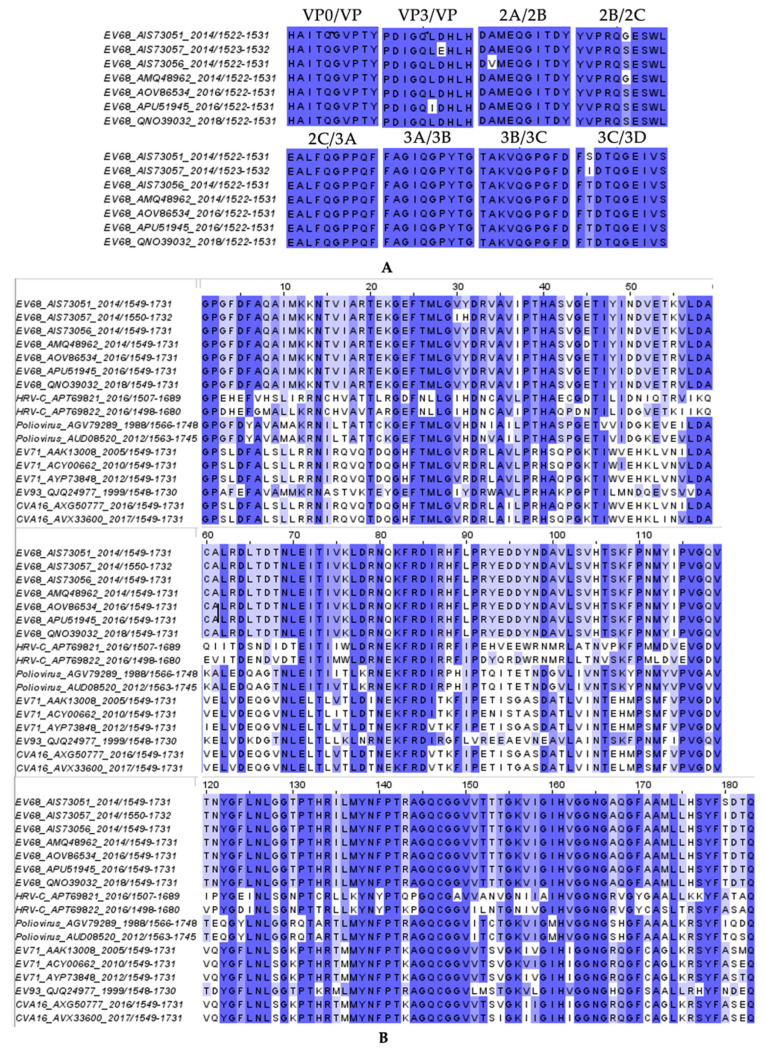
The conservation of amino acid sequences for 3C protease and viral polyprotein cleavage sites: (**A**) cleavage site sequences in EV68 strains and (**B**) 3C protease sequences across picornaviruses, highlighting the conserved regions with darker shades.

While there are currently no U.S. Food and Drug Administration (FDA)-approved therapies against EV68, or any other non-polio enterovirus [3,15,16], previous studies on similar viruses, such as human rhinovirus (HRV) and enterovirus-A71 (EV71), demonstrated 3C protease to be a potential antiviral target [17,18,19]. Over the past two decades, various 3C protease inhibitors have been synthesized and evaluated [15,16,17,20,21,22,23], with irreversible covalent inhibitors such as rupintrivir (AG7088), AG7404, and reversible covalent inhibitor GC-376, being tested against a variety of picornaviruses [15,16,17,19,21,22,23,24,25,26,27,28]. Among strains of EV68, the amino acid sequences of 3C protease and its cleavage sites are highly conserved (Figure 1A) [29]. Even beyond EV68, the 3C protease is highly conserved in other enteroviruses and members of the Picornaviridae family (Figure 1B). Thus, targeting the 3C protease has promise to produce robust, potentially pan-enteroviral 3C protease inhibitors. Direct-acting antivirals (DAAs) target essential processes, such as cleavage by the viral protease, in the viral lifecycle [3,15,16,27,30]. The 3C protease cleaves eight sites within the viral polyprotein, with a preference for a glutamine–glycine (QG) motif at the P1/P1′ positions, a preference distinct from host cysteine proteases [1,17]. Inhibitors targeting viral proteases have proven to be effective antivirals that can advance to clinical use, as seen recently with nirmatrelvir for the SARS-CoV-2 3C-like main protease [30]. In addition to 3C-like protease inhibitors, 3C protease inhibitors continue to be explored as potential antiviral drugs. 

Strategies to design inhibitors that minimize the emergence of drug resistance remain a critical challenge to develop robust DAAs. Due to the low fidelity and lack of proofreading mechanism of the RNA-dependent RNA polymerase of EV68, a high rate of mutations (5–12 × 10^−3^ substitutions per nucleotide per year) [31,32] can be introduced throughout the translated polyprotein, leading to possible resistance mutations. We have previously characterized the substrate envelope for the EV68-3C protease [33], which allows structural analysis to predict potential sites of drug resistance for a given inhibitor. Inhibitors extending beyond the substrate envelope are more susceptible to drug resistance mutations, while inhibitors that fit within this volume exhibit higher barriers to resistance, as we have shown for other viral proteases [34,35,36]. When inhibitors fit within the substrate envelope, mutations that negatively affect inhibitor binding simultaneously impair the protease’s ability to recognize substrates, decreasing the likelihood of viable resistance mutations [37,38,39,40]. We have previously determined the substrate envelope and structurally analyzed inhibitor binding to viral proteases of HIV-1, HCV, and SARS-CoV-2 [35,41,42,43]. The structural determination by protein crystallography of various 3C and 3C-like inhibitors bound to the EV68-3C protease will provide insights into potential sites of resistance mutations.

Here, we measured the potency of various 3C and 3C-like protease inhibitors against EV68-3C protease and structurally analyzed inhibitor binding at the active site. The co-crystal structures of EV68-3C protease we determined with inhibitors (rupintrivir, AG7404, and GC-376) provide insights into potential sites of resistance mutations. Through the analysis of these structures, along with previously reported structures, we hereby uncover well-conserved structural motifs that present opportunities for further inhibitor modifications. Our analysis elucidates additional opportunities for the design of potentially potent and robust protease inhibitors against EV68.

## 2. Materials and Methods

### 2.1. Expression and Purification of EV68-3C Protease

Cleavable His-tagged EV68-3C protease (WT and C147A mutant) with an embedded thrombin cleavage site was cloned into a pET28-a vector and expressed and purified by previously published methods [15]. Non-cleavable His-tagged EV68-3C protease was cloned into a pET21-a vector. Each vector was transformed into Rosetta BL21(DE3) *E. coli* cells using standard techniques. Overnight cultures in TB + kanamycin media were grown from single colonies. Each culture was used to inoculate 6 × 1 L cultures in TB, supplemented with 0.4% glycerol and 100 µg/mL ampicillin. These cultures grew in Fernbach flasks at 37 °C while shaking at 180 rpm, until the OD_600_ reached approximately 0.75, at which point the temperature was reduced to 18 °C, 1 mM IPTG was added, and the culture was left to grow overnight. Cell pellets were resuspended in Buffer 1 (25 mM Tris-HCl pH 8.0, 300 mM NaCl, 10 mM imidazole) prior to lysis by three passes through a cell disruptor/homogenizer at 80 psi. Cell lysate was then clarified by centrifugation at 25,000× *g* for 1 h. A 5 mL His-Trap crude FF column (Cytiva, Marlborough, MA, USA) was pre-equilibrated with Buffer 1 using an AKTA FPLC. The clarified lysate flowed through the column via a peristaltic pump. The His-tagged protein was slowly eluted over a linear gradient from Buffer 1 to Buffer 2 (25 mM Tris-HCl pH 8.0, 300 mM NaCl, 250 mM imidazole) using an AKTA FPLC. The presence of non-cleavable His-tagged EV68-3C protease in the elution peak was confirmed by SDS-PAGE. The protein was concentrated to approximately 2 mL prior to purification via gel filtration chromatography on a Superdex 75 16/60 column pre-equilibrated with Buffer 3 (26 mM Tris-HCl pH 8.0, 200 mM NaCl, 5 mM dithiothreitol (DTT), and 10% glycerol (*w*/*v*)). Fractions in the eluted peak were verified with SDS-PAGE, pooled, concentrated, flash-frozen in liquid nitrogen, and stored at −80 °C unless used immediately for crystallization.

### 2.2. Protein Crystallization

Purified non-cleavable His-tagged EV68-3C protease and inhibitors rupintrivir and GC-376 from MilliPore-Sigma and AG7404 from MedKoo were utilized in protein crystallization. The crystals used for seeding were grown by thawing 10 mg/mL of protein on ice and diluting to 5 mg/mL in SEC Buffer (25 mM HEPES pH 7.4, 200 mM NaCl, and 10% glycerol (*w*/*v*)). Crystals were grown using 24-well, pre-greased, VDX hanging-drop trays (Hampton Research Corporation, Aliso Viejo, CA, USA) at various protein-to-precipitant ratios (1 µL:2 µL, 2 µL:2 µL, and 3 µL:2 µL) with previously published conditions [15]. The crystals grew over one week and, due to their small size, were used for seeding. Larger crystals were grown with seeding and by thawing 10 mg/mL of protein on ice, then diluting to 7 mg/mL. These larger crystals were grown using 24-well, pre-greased, VDX hanging-drop trays (Hampton Research Corporation) at a protein-to-precipitant ratio (2 µL:2 µL with seeding, and 2 µL:2 µL without seeding) with optimal conditions. Crystal growth took place at room temperature and required one week to obtain diffraction quality crystals. Prior to complex formation, the protein was centrifuged at 13,000× *g* for two minutes at 4 °C to remove the insoluble particulates that may promote aggregation and hinder crystal growth. Inhibitor complexes with rupintrivir or GC-376 were formed by incubating EV68-3C with 5-fold molar excess of the inhibitor, while the AG7404 inhibitor complex was formed by incubating EV68-3C protease with 3-fold molar excess due to precipitation at a higher molar excess. All complexes were incubated on ice for two hours. To limit vibration, crystallization trays were placed on foam padding.

### 2.3. Data Collection and Structure Determination

X-ray diffraction data were collected at 100 K. Co-crystals were soaked in cryogenic solutions made by supplementing the exact precipitant solutions with 15% ethylene glycol. Crystallographic data were collected at the Brookhaven National Laboratory NSLS-II Beamline 17-ID-2 (FMX). The diffraction intensities were automatically indexed, integrated, and scaled using XDS [44]. All structures were determined using molecular replacement with PHASER [45]. Model building and refinement were performed using Coot [46,47] and Phenix [48,49]. The geometry (dihedral angels and bond lengths) for all inhibitors was optimized with Gaussian (version 16). The reference model used to solve various inhibitors bound to EV68-3C protease by molecular replacement was PDB ID: 3ZVE [15]. Prior to molecular replacement, the model was modified by removing all water, buffer, and cryogenic molecules, as well as any small molecule inhibitors in the active site. To minimize reference model bias, 5% of the data were reserved to calculate R_free_ [50]. The X-ray data collection parameters and refinement statistics are presented in Table 1.

### 2.4. Inhibitor Modeling and Molecular Dynamics Simulations

Prior to molecular dynamics (MD) simulations, inhibitors without crystal structures in 3C proteases were modeled in Schrödinger Suite [51] software Maestro (version 12.2.012) based on a high-resolution crystal structure of EV68-3C protease with rupintrivir (PDB ID: 7L8H) [52]. Each inhibitor was prepared via the Protein Preparation Wizard within Maestro, as previously described [53,54]. Missing side chains were added through Prime [55], hydrogen atoms were added to the structure, protonation states were determined via PROpKA [56], and the hydrogen bonding network was then optimized. Finally, a restrained minimization was performed using the OPLS3 force field [57] within an RMSD of 0.3 Å to minimize the structure prior to simulation. The prepared systems were placed in a cubic TIP3P explicit water box measuring 25 Å on each side. MD simulations were carried out as previously described [54] using Desmond within the Schrödinger Suite [58]. The system was neutralized, and 0.15 M salt was added using sodium cations and chloride anions. The OPLS3 force field was used to parametrize the substrate and protein. All crystallographic waters were retained during structure minimization and the following MD simulation. Prior to starting the simulation, the solvated system was minimized using a gradual, 21-step procedure, as described previously [33]. Triplicates of 150 ns simulations for EV68-3C protease with each modeled inhibitor and randomized starting velocities were performed [53,54]. The root mean square deviation (RMSD) and root mean square fluctuations (RMSFs) of alpha carbon atoms were calculated using tools within the Schrödinger Suite as well as through previously described custom Python scripts on Github [59].

### 2.5. Structural Analysis: Hydrogen Bonds and Van Der Waals Interactions

For all co-crystal and modeled structures, intermolecular interactions between the inhibitor and protease were analyzed using custom Python scripts, as previously described [59]. Hydrogen bonds were displayed using the show contacts PyMOL Plugin with default parameters, according to which the bond angle is between 63° and 180° and the distance is less than 4.0 Å for any bond and 3.6 Å for an ideal hydrogen bond between the proton and heavy atom. A further analysis was conducted on simulation trajectories to determine the high-frequency hydrogen bonds between the inhibitor and protease. Van der Waals interaction energies between the inhibitor and protease were estimated using a simplified Lennard–Jones potential, as previously described [60].

### 2.6. Enzyme Activity Assay to Determine K_M_

To assess enzyme activity, a fluorogenic substrate cleavage assay was developed for EV68-3C protease, based on previously reported protease assays [61]. In a light-blocking, flat-bottom, 96-well plate, a 10-mer amino acid substrate, corresponding to the 2C/3A natural cleavage site (Supplemental Figure S1), with a DACBYL/EDANS FRET pair (GenScript) solvated in 3% DMSO and a buffer (50 mM HEPES pH 7.5 and 100 mM NaCl) was serially diluted (3/4) from 55 μM to 0 μM. Using a PerkinElmer EnVision plate reader, 5 μL aliquots of EV68-3C protease were dispensed onto the 96-well plate at a final concentration of 10 nM. The fluorescence readout was measured with an excitation at 340 nm and an emission at 496 nm. The FRET inner filter effect was not observed at the substrate concentrations used to optimize this assay. All assays were repeatable and contained a 0 μM substrate control group. All data were analyzed via Prism (version 9.4.1). The determined K_M_ for EV68-3C protease was consistent with the previously reported value with the same substrate (see Supplemental Figure S1).

### 2.7. Enzyme Inhibition Assay to Determine IC_50_

To assess the potency of various 3C and 3C-like inhibitors, the fluorogenic enzyme assay was utilized to determine the IC_50_ with Prism (version 9.4.1). Each inhibitor, serially diluted (1/2) across three orders of magnitude (typically 500 nM to 500 pM or 10 μM to 10 nM), was co-incubated with 25 nM of EV68-3C protease for 60 min prior to the addition of the substrate. The substrate was the same 10 mer amino acid corresponding to the 2C/3A natural cleavage site, with a DABCYL/EDANS FRET pair (GenScript). The co-incubated inhibitor and protein were solvated in 3% DMSO and buffer (50 mM HEPES pH 7.5 and 100 mM NaCl). The substrate was dissolved in 6% DMSO at a concentration of 120 μM. Aliquots of 5 μL substrate were dispensed using a PerkinElmer Envision plate reader into the 96-well plate at a final concentration of 10 μM. The fluorescence readout was measured with excitation at 340 nm and emission at 496 nm. All assays were repeated in triplicate and contained a control with no inhibitor. All data were analyzed via Prism (version 9.4.1).

## 3. Results

### 3.1. Characterization of 3C and 3C-Like Inhibitors for Activity Against EV68-3C Protease

Various 3C and 3C-like inhibitors have been reported for viral proteases, but the potency against EV68-3C protease has not been directly measured in enzymatic assays. Inhibitors were selected for testing based on prior inhibition of other 3C or 3C-like proteases and commercial availability, and they were prescreened for stability within the EV68-3C protease active site by MD simulations. These selection criteria yielded six promising compounds to experimentally test. The inhibitors (Supplemental Figure S2) were predicted to bind within the active site of EV68-3C protease, and the inhibitor ensitrelvir was included as a negative control as it was unstable within the active site during MD simulations (Supplemental Figure S3). Rupintrivir, AG7404, GC-376, and PF-00835231 showed inhibition against EV68-3C protease, while there was no observable inhibition with nirmatrelvir or ensitrelvir in this assay (Table 2). Rupintrivir demonstrated the highest potency with the IC_50_ value in the single-digit nanomolar range, followed closely by AG7404. Only nirmatrelvir behaved contrary to our expectations, as MD simulations suggested that nirmatrelvir would be stable within the active site, but no inhibition was observed experimentally, even at concentrations as high as 10 μM. While nirmatrelvir proves extremely potent against SARS-CoV-2 Mpro, a 3C-like protease, with single-digit nanomolar potency [62], the lack of inhibition against EV68-3C protease may be due to the unique geometry of the backbone-integrated bicyclic proline at the P2 position compared to other 3C protease inhibitors. From these data, the shared scaffold of rupintrivir/AG7404 appears to be well accommodated within the active site of EV68-3C protease.

### 3.2. Co-Crystal Structures of EV68-3C Protease with the Inhibitor Bound Within the Active Site

**Figure 2:**
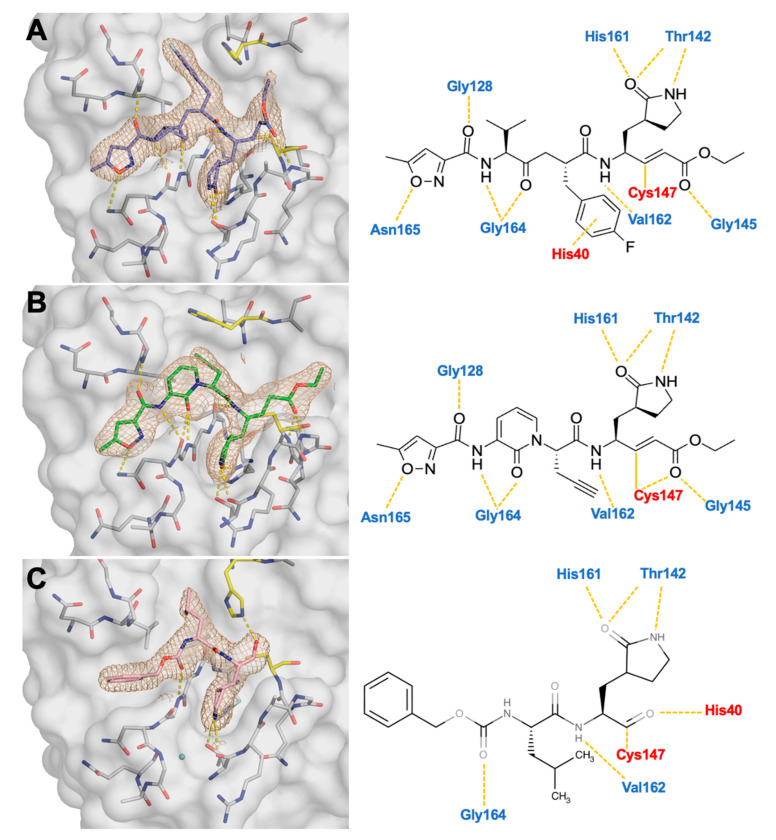
Co-crystal structures of EV68-3C protease with inhibitors: (**A**) irreversible covalent inhibitors rupintrivir (slate blue) and (**B**) AG7404 (green) and (**C**) reversible covalent inhibitor GC-376 (pink). On the left panels, the protease is depicted as a gray surface while the inhibitor is represented as a colored stick within the electron density (2F_0_-F_C_ map) and hydrogen bond interactions (yellow dashed lines) shown with the surrounding protease residues (gray sticks). The catalytic dyad residues (H40 and C147) are highlighted in yellow. The chemical structures of the bound inhibitors and hydrogen bonds with the EV68-3C protease residues are displayed on the right.

While co-crystal structures of 3C protease inhibitors exist, many of these inhibitors have yet to be characterized with EV68-3C protease. We performed extensive co-crystallization trials with the 3C inhibitors that showed activity against EV68-3C in the enzymatic assays and achieved diffraction-quality crystals with rupintrivir, AG7404, and GC-376 (Table 1). These structures were obtained with an EV68-3C protease construct with a non-cleavable C-terminal His-tag promoting crystal lattice packing [15]. This packing of the protein leaves the active site unobstructed for ligand binding. The resulting structures are all the same space group (P3 21 1), had a clearly defined electron density for the entire inhibitor, and diffracted to sub 2 Å, facilitating direct comparison (Figure 2). While AG7404 or GC-376 had not been crystallized with EV68-3C protease before, a structure of rupintrivir bound to an alternative construct without the His-tag had been reported by us previously (PDB ID: 7L8H) [52]. When comparing these two rupintrivir-bound structures, the protease interactions with the inhibitor were highly similar, but minor differences in crystal contacts were noted. Therefore, the His-tag and crystal packing did not alter inhibitor binding. Furthermore, each structure informs on the hydrogen bond interactions between EV68-3C protease residues and the inhibitor within the active site. Within the active site, rupintrivir and AG7404 exhibited identical hydrogen bonds from P4 to P1′, with one additional hydrogen bond formed between AG7404 and C147 backbone nitrogen (Figure 2). GC-376, a smaller inhibitor, formed similar hydrogen bonds, with several residues found in all three inhibitors. Across all three structures, interactions between the inhibitor and Thr142, His161, Val162, and Gly164 were conserved. These interactions were all required for EV68-3C protease to bind viral substrates [33]. In addition to these conserved hydrogen bond interactions, we noticed two additional structural features in the co-crystal structures, involving the catalytic His40 and the S1 pocket, which we further analyzed to identify the key interactions in inhibitor binding.

### 3.3. Crystal Structures Reveal the Conformational Flexibility of His40 to Accommodate Inhibitors Within the Active Site

The position of the catalytic His40 varied considerably among these inhibitor-bound crystal structures even though the interactions between the protease and the bound inhibitors were overall conserved. The superposition of the three inhibitor-bound structures we solved demonstrated that the inhibitors occupied the same pockets within the active site of EV68-3C protease (Supplemental Figure S4). The observed conformational flexibility of His40 was likely due to differences between inhibitor P2 moieties. At P2, GC-376 lacked the aromaticity in rupintrivir (Figure 2), and the isobutyl group fit into the defined S2 pocket without disrupting His40 from the substrate-bound position we had previously observed in the 3B3C substrate-bound EV68-3C co-crystal structure (PDB ID: 9AX9) [33]. The conformation of the catalytic His40 within the rupintrivir co-crystal structure recapitulated our previously published structure (PDB ID: 7L8H) [52], as the P2 fluorinated phenyl ring formed a strong pi-stacking interaction with His40 (Figure 2A). At P2, AG7404 lacked the fluorinated phenylalanine ring of rupintrivir and was unable to pi-stack on the catalytic His40. In this structure, we observed the catalytic His40 in an alternative conformation, where the side chain was rotated furthest away from the substrate-bound position (Figure 2C). This His40 conformation was also seen in the previously solved SG-85 inhibitor-bound EV68-3C protease structure (PDB ID: 3ZVF) [15]. While these alternative His40 conformations had been observed in prior studies, they were thought to have been due to the pH of the crystallization conditions [15]. However, all three of these co-crystal structures were obtained under the same conditions, producing three distinct His40 conformations. The conformational flexibility of His40 could provide additional opportunities to design inhibitors with more specialized P2 groups.

### 3.4. Stabilization of the S1 Pocket and a Key Structural Water Molecule in Ligand-Bound EV68-3C Structures

**Figure 3:**
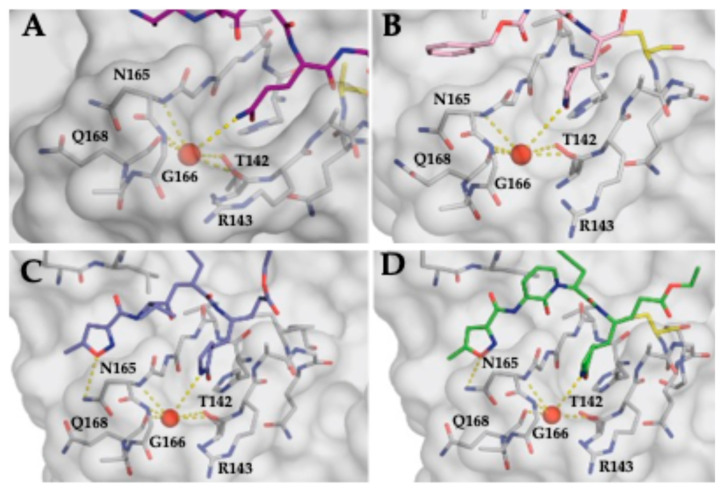
Coordination of an ordered water molecule within the S1 pocket of EV68-3C protease in ligand-bound crystal structures: (**A**) 3B3C peptide (purple sticks) PDB IDs: 9AX9 and (**B**–**D**) inhibitors (GC-376 in pink, rupintrivir in slate blue, and AG7404 in green, respectively) bound within EV68-3C protease. The hydrogen bonds between the water (red sphere) and surrounding EV68-3C protease residues (Thr142, Asn165, and Gly166) are depicted as dashed lines.

The EV68-3C protease co-crystal structures suggest a conserved motif required for stable ligand binding involving a structural water molecule. We previously identified conformational changes at the active site in substrate-bound structures compared to apo protease structures, including an ordered water molecule which not only stabilized the P1 glutamine but also coordinated capping of the S1 pocket through a hydrogen bond network [33]. The conformational changes in EV68-3C protease we observed for substrate binding were also present in inhibitor-bound structures. For the inhibitor-bound structures (PDB ID: 8W3C, 8W3M, and 8W3T) and the substrate-bound structure (PDB ID: 9AX9) [33], a water molecule coordinated the stabilization of S1 pocket residues (Figure 3). Specifically, the Thr142 backbone oxygen and sidechain hydroxyl, the Asn165 backbone nitrogen, the Gly166 backbone nitrogen, and the Gln168 backbone oxygen were all coordinated by this structural water molecule (Figure 3). All other available structures of EV68-3C protease bound to an inhibitor (PDB ID: 7L8H, 3ZVA-G, 3ZV9) contained the structural water molecule capping the S1 pocket [15,52]. For all apo EV68-3C structures (PDB ID: 8FL5, 3ZV8), the water was absent (Supplemental Figure S5) [15,33]. This finding suggests that the structural water molecule plays a key role in ligand binding for EV68-3C protease.

### 3.5. Substrate Envelope Analysis of Inhibitor Co-Crystal Structures Predicts Potential Sites of Resistance

**Figure 4:**
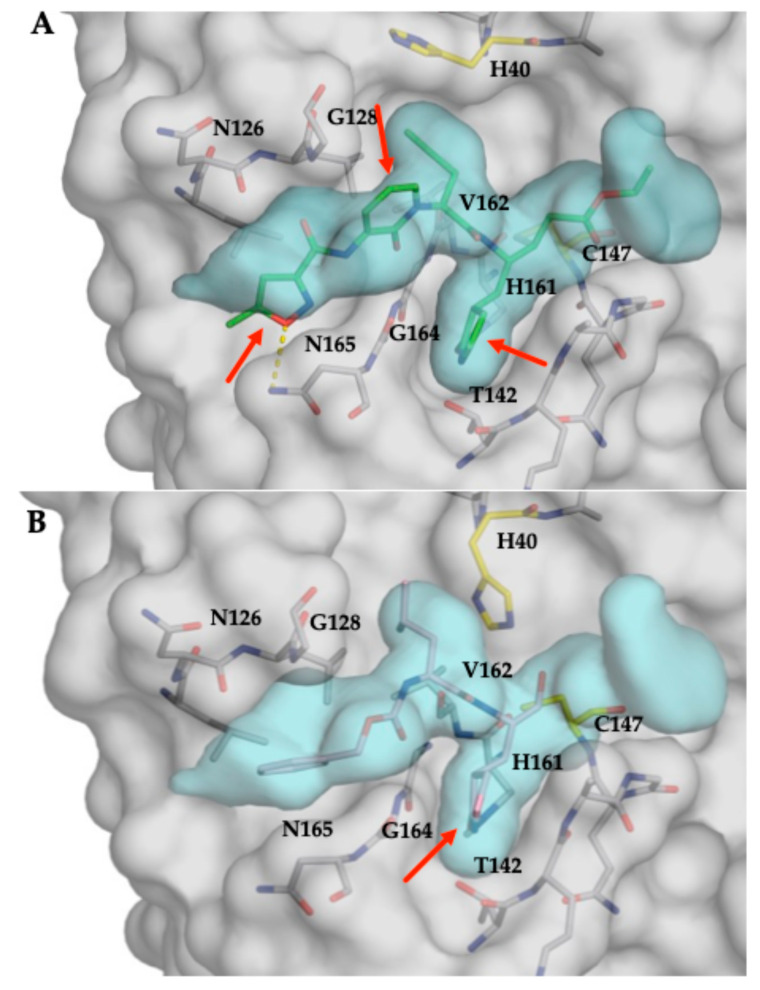
Fit of inhibitors within the EV68-3C protease substrate envelope: inhibitors (**A**) AG7404 (green sticks) and (**B**) GC-376 (pink sticks). The protease is shown as a gray surface with residues around the inhibitor represented as sticks (gray) and labeled. The inhibitor from the co-crystal structures has been superimposed onto the substrate envelope (cyan volume). Red arrows highlight regions of the inhibitor protruding from the substrate envelope.

These co-crystal structures, in conjunction with the EV68-3C substrate envelope we previously determined [33], enable us to identify potential sites of resistance against AG7404 and GC-376 in the EV68-3C protease. AG7404 (PDB ID: 8W3M) (Figure 4A) and GC-376 (PDB ID: 8W3T) (Figure 3B) were analyzed for fitting within the EV68-3C substrate envelope. Both inhibitors protruded out of the substrate envelope at the S1 position and AG7404 also extended beyond the envelope at the S2/S3 regions and S4 pocket. Within the S1 pocket, the g-lactam ring extended beyond the substrate envelope into the solvent. Based on this observation, mutations at Gly166 or Ala144 could introduce a steric bulk, blocking the protruding P1 moieties and decreasing the inhibitor’s ability to bind without impacting substrate binding. While mutations at these residues were not observed in about 1300 fully sequenced EV68 genomes [29], they may emerge under the selective pressure of inhibition. Between the S2 and S3 pocket, AG7404 contains an aromatic ring incorporated into the peptidomimetic backbone structure that protrudes outside of the substrate envelope. In this region, the mutation of Gly128 into a larger amino acid has the potential to interfere with inhibitor binding and confer resistance. As AG7404 is an analog of rupintrivir, this compound shares many structural similarities and protrudes in the same regions [33], highlighted at the S4 pocket, where the isoxazole ring forms the same hydrogen bond contact with Asn165. At the S4 pocket, the mutation of Asn165 into a larger residue could preclude binding of the inhibitors, while other mutations could remove the potential for hydrogen bond formation, potentially decreasing inhibitor binding. Furthermore, this additional hydrogen bond formation, not conserved in viral substrate interactions, has been implicated in resistance against rupintrivir in HRV-3C protease [24]. For rupintrivir (PDB ID: 8W3C), the results mirrored the potential sites of resistance predicted for our previous rupintrivir co-crystal structure (PDB: ID 7L8H) [33,52]. From these data, we predict that GC-376 would have a higher barrier to resistance compared to rupintrivir or AG7404. Thus, using the substrate envelope, we identified potential sites for resistance and moieties of current inhibitor scaffolds to preemptively avoid resistance.

## 4. Discussion

With the continued emergence of new EV68 strains in the United States and the long-term neurological sequalae associated with the severe disease burden of infections, a better understanding of current inhibitors targeting the conserved 3C protease is needed [5,9,10,13,32]. While 3C inhibitors have been previously reported [15,19,27,63,64,65,66,67,68], there are no FDA-approved antivirals to address non-polio enteroviral species. Moreover, the design of inhibitors typically does not consider possible resistance mutations. Here, we have determined the potency of various 3C and 3C-like inhibitors against EV68-3C protease and the co-crystal structures of EV68-3C protease with those inhibitors, gaining insight into their binding, and utilized the EV68-3C protease substrate envelope [33] to further our understanding of inhibitor susceptibility to potential drug resistance mutations. These results also identified conserved structural elements in ligand-bound structures, exposing potential regions to modify current 3C inhibitors to aid in the design of robust DAAs.

The substrate envelope analysis applied to the co-crystal structures of EV68-3C protease highlights potential sites of resistance and regions where inhibitor modifications can be introduced to minimize susceptibility to resistance mutations. Inhibitors extending beyond the confines of the envelope represent opportunities for resistance to arise [34,35,36]. The interaction of inhibitors with residues at the S1 pocket (Ala144 and Gly166), between S2 and S3 (Gly128), and the isoxazole ring at the S4 pocket (Asn165) raise concern for resistance mutations. While there are no sequences of circulating strains with mutations at these residues, mutations can emergence under repeat exposure to inhibitors. This concern is exemplified by previous studies that demonstrated a 7-fold (V104I) and 15-fold (N165T, A103V, and E3G) decrease in potency when mutations arise in HRV-3C protease against rupintrivir [22,24]. From our structural analysis, we predict that EV68 strains with similar mutations would likely demonstrate some level of resistance toward these inhibitors. Understanding potential sites of resistance will assist in the development of long-lasting and robust EV68-3C inhibitors, and the structural analysis we present here provides additional information to further aid inhibitor design.

The inhibitor-bound crystal structures of EV68-3C protease we have determined revealed certain patterns and structural elements common between all ligand-bound structures that, when targeted by inhibitors, could maintain a high barrier to resistance mutations. These primarily involve the catalytic His40 and the structural water molecule located within the S1 pocket. This water is also present in inhibitor-bound 3C protease structures of other enteroviruses and related viruses from the Picornaviridae family, including EV71, EV93, poliovirus, human rhinovirus type C (HRV-C), and coxsackievirus-A-16 (CVA-16) (PDB IDs: 4GHT, 4GHQ, 3Q3Y, 3Q3X, 4DCD, 1L1N, 6KU8, 6KU7, and 3SJI, 3SJ8) [17,23,69,70,71,72] (Supplemental Figure S5), and all available ligand-bound EV68-3C structures (Supplemental Figure S5). This conservation suggests that this structural water molecule plays a crucial role in ligand binding for 3C proteases and may represent additional opportunities to design pan-viral 3C inhibitors. One strategy to leverage the conserved water molecule in the S1 pocket might be to design modified P1 groups that mirror the hydrogen bond network of this water molecule, primarily interacting with the backbones of Asn165, Gly166, and Gln168, supplanting the water and coordinating the S1 cap. With the cavity of the S1 pocket itself, the only residue side chain present is Thr142, which is conserved across all strains analyzed (Figure 1 and Figure 2) and all sequenced genomes of EV68 [29]. This concept is further supported by the fact that any mutation at Gly166 would introduce bulk, which is not found in any known strains [29]. While this alternative P1 moiety would extend beyond the substrate envelope, mutations are less likely to provide an evolutionary advantage under the selective pressure of inhibition. We expect that the changes in this region that negatively affect inhibitor binding would simultaneously impair the protease’s ability to recognize and bind substrates, decreasing susceptibility to resistance mutations [37,38,39,40]. Another structural feature we observed in the co-crystal structures was the conformational flexibility of His40, which adopted various conformers in the crystal structures. Rotation of the His40 side chain away from the position in a substrate-bound conformation creates additional space behind P1’, which might be exploited for inhibitor expansion with low susceptibility to resistance mutations. While this space behind P1′ is outside of the substrate envelope, mutations in this area would not be tolerated, as mutating the catalytic His40 (or immediate neighbors) would simultaneously prevent the proteolytic cleavage of substrates.

EV68, considered by the NIH as a pathogen of concern, continues to be a global health issue and remains without FDA-approved antiviral drugs for treatment. Our structural analysis here provided further insights into inhibitor binding at the EV68-3C protease active site and possible sites of resistance mutations for current inhibitors. We have identified the conserved water within the S1 pocket and the conformational flexibility of His40 as structural features that might be leveraged in drug design. As the 3C protease is well conserved across the Picornaviridae family, these strategies can be applied to other viruses, such as EV71, EV93, poliovirus, human rhinovirus, and coxsackievirus. While we previously showed that the emergence of resistance can be minimized with inhibitors leveraging the evolutionary constraint of substrate specificity [34,35,36,37,38,39,40,41,42,43], these structural analyses provide further insights for the rational design of therapeutics resilient to drug resistance mutations of EV68-3C protease.

## Figures and Tables

**Table 1:** Crystallographic data collection and refinement statistics for inhibitor-bound EV68-3C protease structures.

Inhibitor PDB ID	AG-7088 8W3C	AG-7404 8W3M	GC-376 8W3T
**Data Collection**			
Location	NLS-II, Synchrotron	NLS-II, Synchrotron	NLS-II, Synchrotron
Resolution Range (Å)	32.12–1.97 (2.04–1.97)	32.16–1.97 (2.06–1.97)	32.07–1.98 (2.05–1.98)
Space Group	P 31 2 1	P 31 2 1	P 31 2 1
a, b, c, (Å)	56.431, 56.431, 170.482	56.491, 56.491, 170.72	56.174, 56.174, 170.629
Alpha, Beta, Gamma (°)	90, 90, 120	90, 90, 120	90, 90, 120
Total Reflections	46,210 (4560)	46,334 (3768)	45,125 (4362)
Unique Reflections	22,964 (2671)	23,046 (2663)	22,473 (2668)
Multiplicity	2 (2.0)	2 (2.0)	2 (2.0)
Completeness (%)	99.29 (99.04)	99.31 (98.80)	99.46 (99.36)
(Average I)/sigma	16 (4.3)	15 (4)	14 (4)
Wilson B-Factor	36.97	37.33	39.51
R_merge_	0.018 (0.094)	0.023 (0.141)	0.029 (0.405)
CC1/2	1.000 (0.993)	0.999 (0.991)	0.999 (0.917)
Refinement *			
R_factor_	0.2131 (0.3553)	0.2056 (0.3918)	0.1993 (0.3378)
R_free_	0.2363 (0.5142)	0.2385 (0.4589)	0.2348 (0.4214)
**RMSD in:**			
Bond Lengths (Å)	0.007	0.008	0.010
Bond Angles (°)	1.03	0.981	1.16
**Ramachandran:**			
Favored (%)	97.81	95.63	97.85
Allowed (%)	2.19	4.37	2.15
Outliers (%)	0	0	0
Rotamer outliers (%)	0	0	0
**B-Factors:**			
Average	46.94	46.53	47.76
Macromolecules	46.19	45.61	47.68
Solvent	53.64	53.46	55.86

* R_factor_ = Σ ||F_o_| − |F_c_||/Σ|F_o_| and R_free_ values were calculated from 5% of reflections, chosen randomly, which had been omitted from the refinement process. The statistics for the highest-resolution shell are shown in parentheses.

**Table 2:** Activity of 3C and 3C-like inhibitors against EV68-3C protease. The half-maximal inhibitory concentration (IC_50_) was determined in enzymatic assays with the mean and SEM (standard error of the mean) reported from three replicates.

Inhibitor	IC_50_ (μM)
rupintrivir (AG7088)	0.0078 ± 0.0006
AG7404	0.0235 ± 0.0011
GC-376	0.194 ± 0.019
PF-00835231	1.8 ± 0.2
nirmatrelvir (PF-07321332)	>10 μM
ensitrelvir	>10 μM

## Data Availability

The data that support this study are available from the corresponding authors upon reasonable request. The crystal structures determined in the current study will be released on publication on the Protein Data Bank (https://www.rcsb.org, accessed on 1 January 2025) with accession codes 8W3C, 8W3M, and 8W3T.

## Supplementary Materials

The following supporting information can be downloaded at https://www.mdpi.com/article/10.3390/v17010075/s1: Figure S1. Enzyme activity assay of EV683C protease; Figure S2. Chemical structures of the 3C and 3C-like inhibitors selected and tested against the EV68-3C protease; Figure S3. Snapshots from MD simulations of inhibitors rupintrivir, AG7404, and GC-376 within the EV68-3C protease’s active site; Figure S4. Crystal structures of various picornavirus 3C proteases, highlighting the structural water molecule within the S1 pocket; Figure S5. Crystal structures of EV68-3C protease in apo and inhibitor-bound forms, highlighting the structural water molecule within the S1 pocket.
