# Supplementary material for: Structural Analysis of Inhibitor Binding to Enterovirus-D68 3C Protease
Source: Viruses. 2025 Jan 8;17(1):75. doi: 10.3390/v17010075 (PMC11768739; doi:10.3390/v17010075)
Supplement: Supplementary file 1 [file viruses-17-00075-s001.zip › viruses-3408373-supplementary.pdf]

SUPPLEMENTARY MATERIALS

Structural Analysis of Inhibitor Binding to EV68-3C Protease

Vincent N. Azzolino, Ala M. Shaqra, Akbar Ali, Nese Kurt Yilmaz, Celia A. Schiffer *

Department of Biochemistry and Molecular Biotechnology, University of Massachusetts Chan Medical School, Worcester, MA, USA
* Correspondence: Celia.Schiffer@umassmed.edu

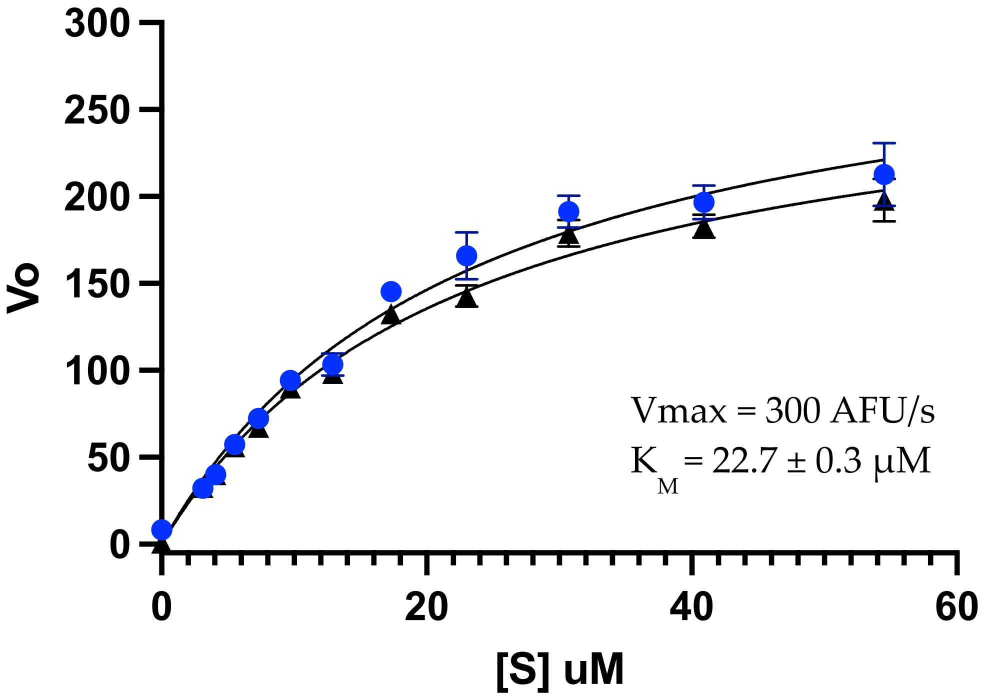

Figure S1. Fluorogenic enzyme activity assay used to determine the K_M of EV68-3C protease for the 2C/3A peptide (EALFQ/GPPGF). 10 nM of EV68-3C protease cleaved the DABCYL and EDANS (**DABCYL-**K-EALFQ/GPPGF-E-**EDANS**) labeled peptide. AFU: arbitrary fluorescent units.

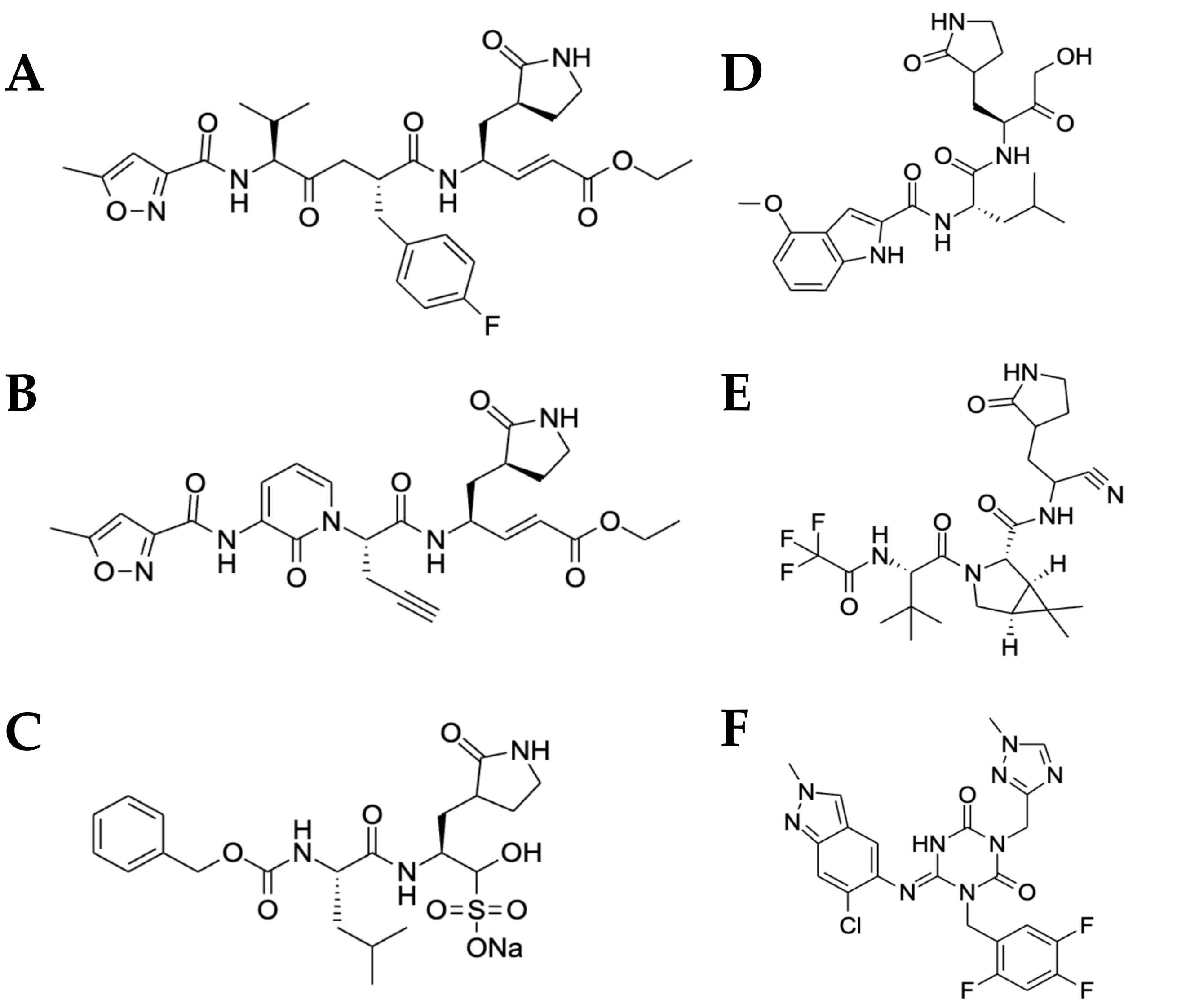

Figure S2. Chemical structures of the 3C and 3C-like inhibitors selected and tested against EV68-3C protease. **A)** Rupintrivir (AG7088) **B)** AG7404 **C)** GC-376 **D)** PF-00835231 **E)** Nirmatrelvir **F)** Ensitrevlir (S-217622).

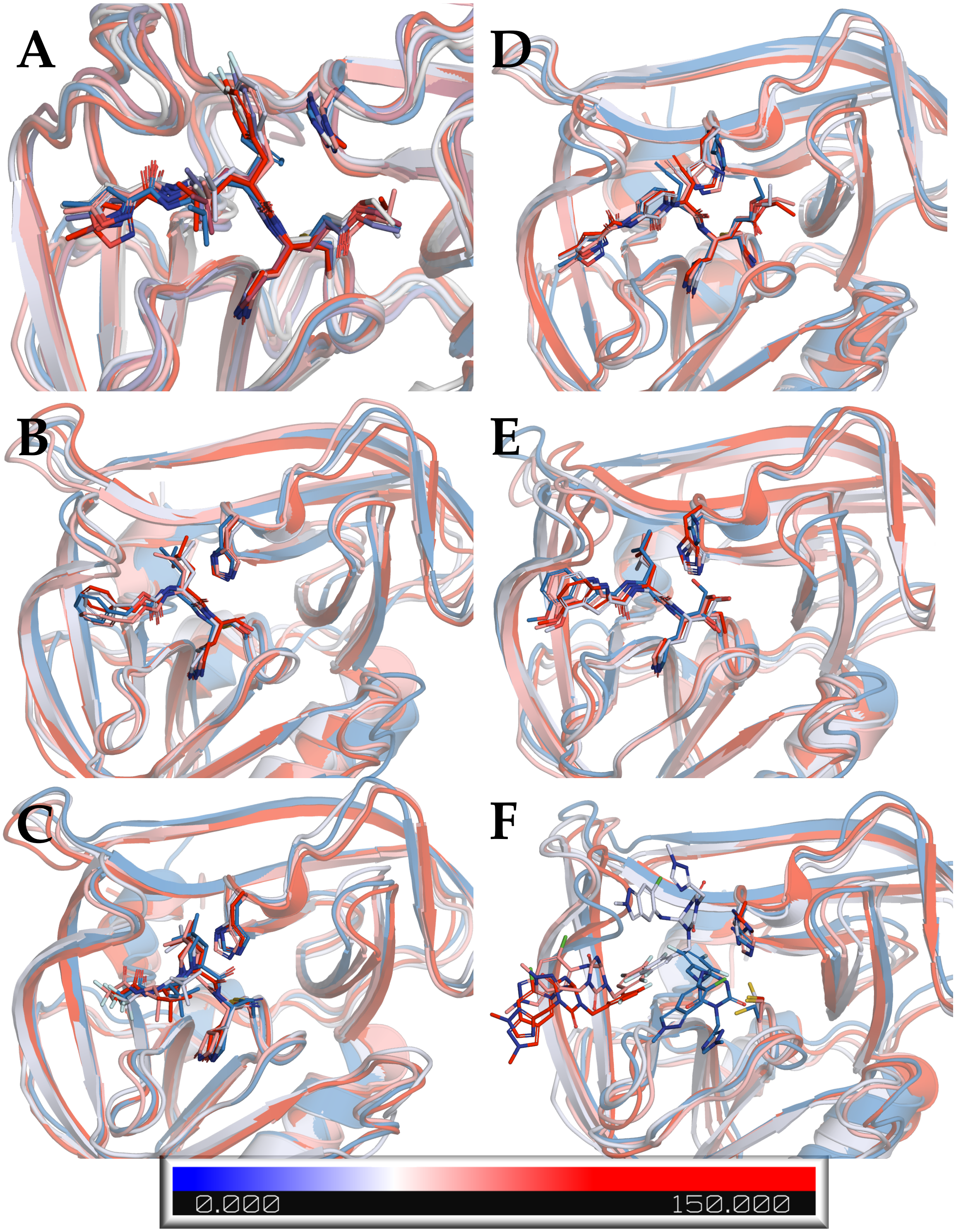

Figure S3. Snapshots from MD simulations of inhibitors bound to EV68-3C protease demonstrating inhibitor position. Blue represents time at 0 ns and red representes the end time point at 150 ns. A) Rupintrivir from co-crystal structure (PDB ID: 7L8H), and modeled inhibitors B) GC-376 C) nirmatrelvir D) AG7404 E) PF-00835231, and F) ensitrelvir at the active site of EV68-3C protease.

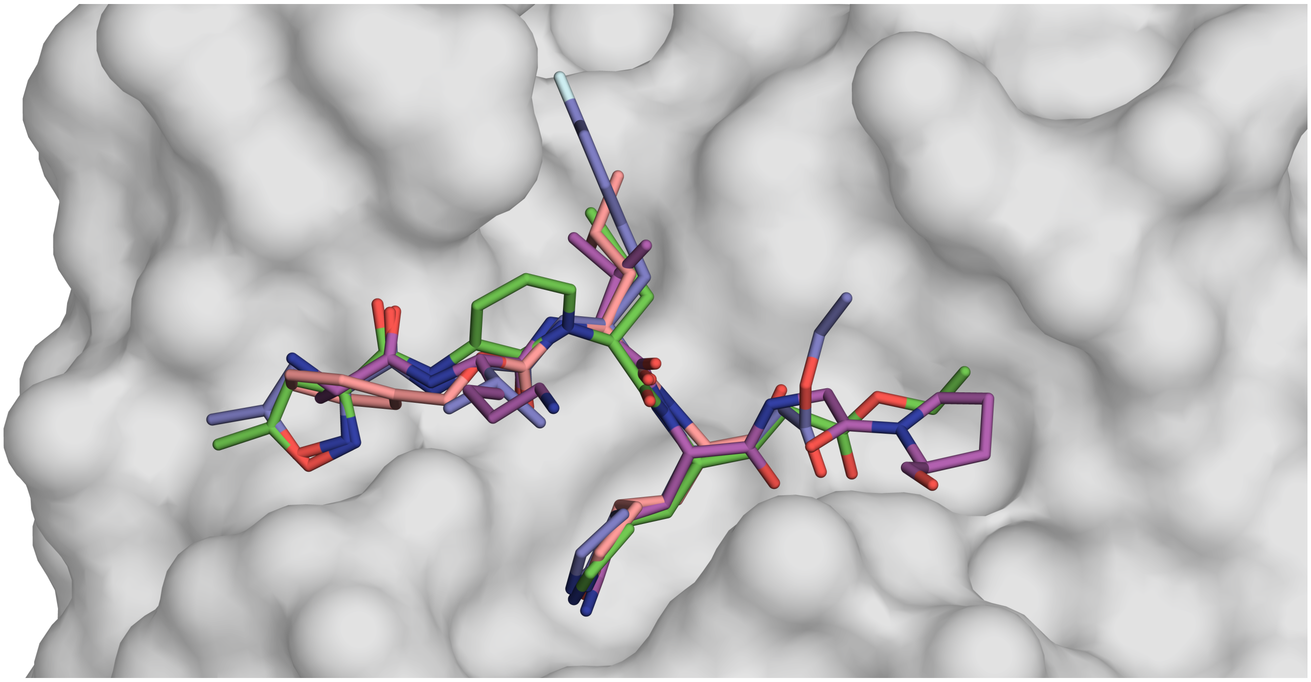

Figure S3. Superposition of inhibitors AG7088 (PDB ID: 8W3C) in slate blue, AG7404 (PDB ID: 8W3M) in green, and GC-376 (PDB ID: 8W3T) in light pink, and the 3B3C peptide in EV68 3C protease (PDB ID: 9AX9) in magenta. The cocrystal structures show that these inhibitors occupy a similar space within the active site of EV68-3C protease.

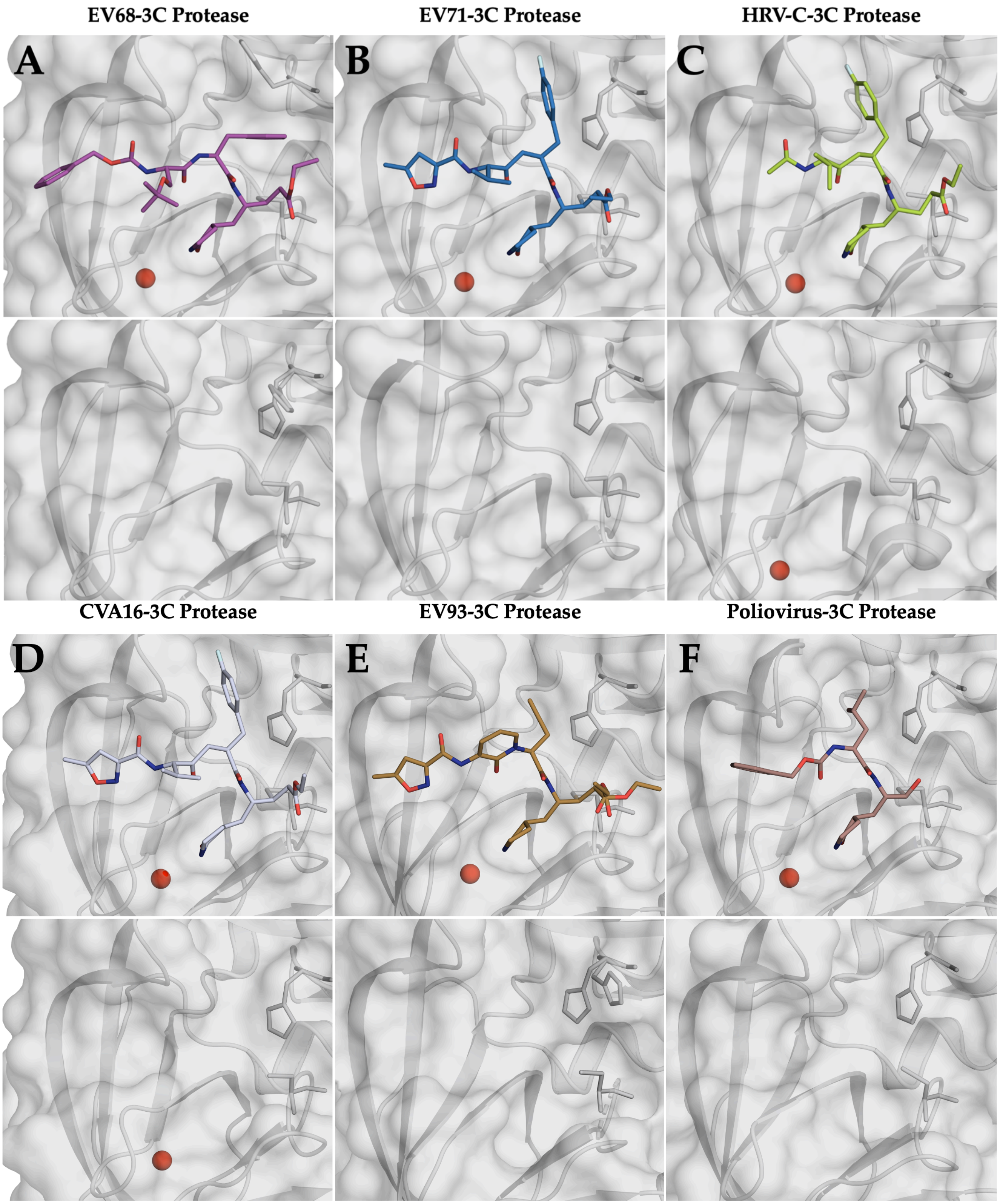

Figure S4. Crystal structures of 3C proteases across various viruses within the picornaviridae family. A conserved structural water (red sphere) exists within all ligand-bound structures but is often absent in apo crystal structures of the same protease. **A)** EV68-3C protease bound to ligand (SG-85) and apo (PDB ID: 3ZVF and 3ZV8). **B)** EV71-3C protease bound to rupintrivir and apo (PDB ID: 4GHT and 4GHQ). **C)** HRV-3C protease bound to rupintrivir (incomplete density) and apo (PDB ID: 6KU8 and 6KU7). **D)** CVA16-3C protease bound to rupintrivir and apo (PDB ID: 3SJI and 3SJ8). **E)** EV93-3C protease bound to AG7404 and apo (PDB ID: 3Q3Y and 3Q3X). **F)** Polio-3C protease bound to a covalent dipeptidyl inhibitor and apo (PDB ID: 4DCD and 1L1N).

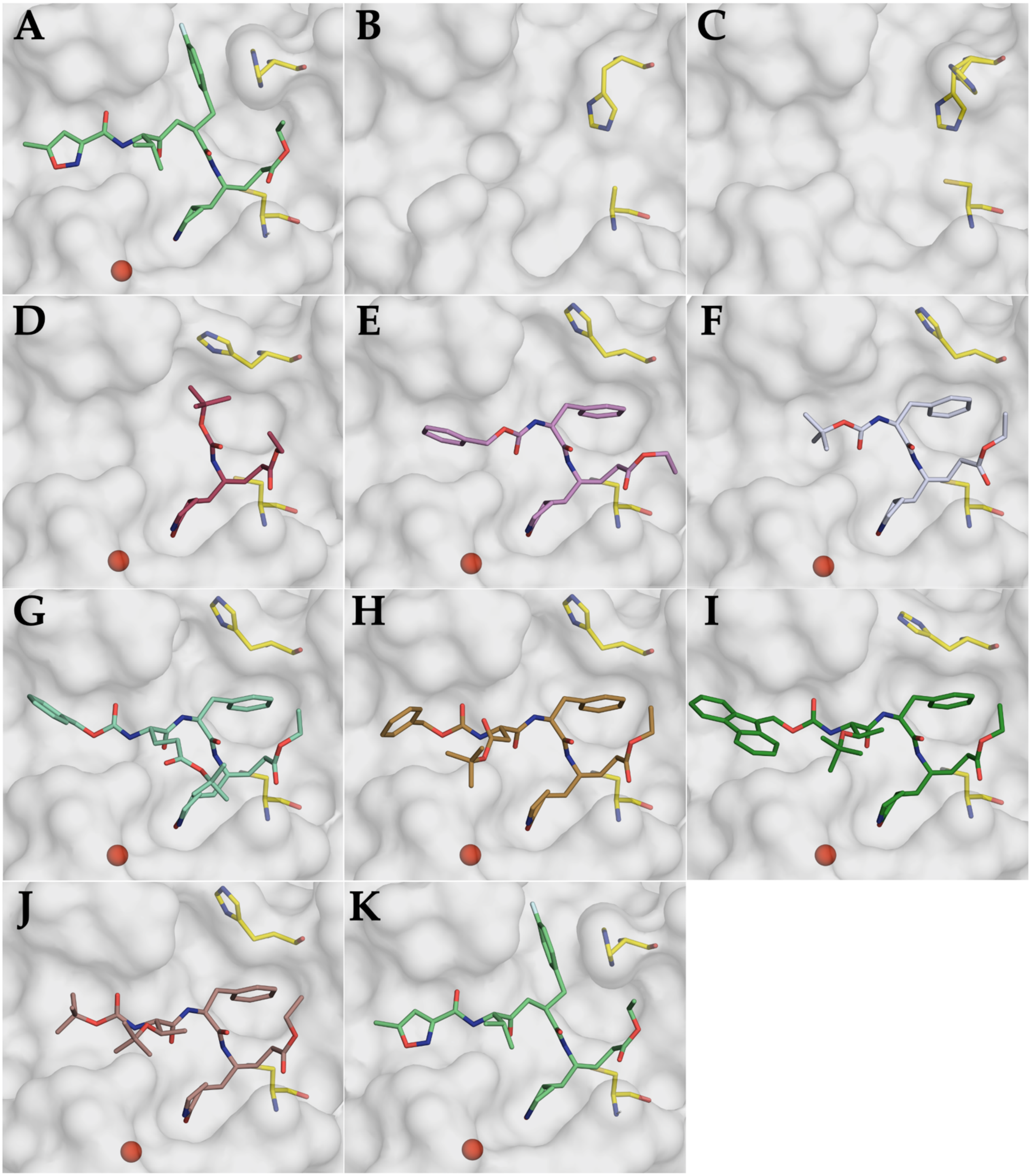

Figure S5. All available crystal structures of apo and inhibitor-bound EV68-3C protease on PDB, with the conserved structural water (red sphere) within all ligand-bound structures; **A)** bound to rupintrivir (PDB ID: 7L8H) **B:** C147A mutant apo structure (PDB ID: 8FL5), **C**) wild-type apo structure (PDB ID: 3AV8). **D-K)** bound to a suite of related peptidomimetic inhibitors (PDB ID: 3ZV9, 3ZVA-E, 3ZVG, 3ZVH).
